# Tuning of Classifiers to Speed-Up Detection of Pedestrians in Infrared Images

**DOI:** 10.3390/s20164363

**Published:** 2020-08-05

**Authors:** Karol Piniarski, Paweł Pawłowski, Adam Dąbrowski

**Affiliations:** Division of Signal Processing and Electronic Systems, Institute of Automation and Robotics, Poznan University of Technology, Jana Pawła 24, 60-965 Poznań, Poland; pawel.pawlowski@put.poznan.pl (P.P.); adam.dabrowski@put.poznan.pl (A.D.)

**Keywords:** pedestrian detection, tuning of object classification, night vision, deep convolutional neural networks, ACF detector

## Abstract

This paper presents an experimental evaluation of real-time pedestrian detection algorithms and their tuning using the proposed universal performance index. With this index, the precise choice of various parameters is possible. Moreover, we determined the best resolution of the analysis window, which is much lower than the initial window. By such means, we can speed-up the processing (i.e., reduce the classification time by 74%). There are cases in which we increased both the processing speed and the classification accuracy. We made experiments with various baseline detectors and datasets in order to confirm versatility of the proposed ideas. The analyzed classifiers are those typically applied to detection of pedestrians, namely: aggregated channel feature (ACF), deep convolutional neural network (CNN), and support vector machine (SVM). We used a suite of five precisely chosen night (and day) IR vision datasets.

## 1. Introduction

More than a half (51%) of pedestrian deaths occur at night (cf., European Union road statistics [1]). This is despite the fact that traffic at night is significantly smaller than that during the day. The main reason for this is poorer visibility and significantly shorter driver’s view range (strongly depending on the cars and streets lighting).

Therefore, in this work we have focused on night-vision automotive systems that detect pedestrians from the car drivers’ perspective in low lighting conditions at night. Most systems make use of infrared (IR) light [2,3,4,5,6,7,8] and can merely support evoking the drivers’ attention or operate in self-decisive manner [2,9,10].

The night-vision systems that detect pedestrians usually perform four main stages: first, the IR image acquisition, second, preparation of the so-called region of interest (ROI), which should cover all areas with pedestrian candidates for further processing, third, segmentation of ROI, which separates objects of interest from the background, and fourth, the object classification, which distinguishes pedestrians from other objects. In our opinion, the main role in effectiveness improvement of the overall system plays optimization of the object classification stage [11].

For classification purposes (not only in the context of pedestrian detection), the baseline approach is the use of a single classifier with a fixed input resolution [3,9,10,12]. In the simplest case, to detect pedestrians of various sizes with a single, fixed size classifier, the scanning window is scaled and shifted through an image and, as a result, all pedestrian candidates must be resized (upscaled or downscaled) to the classifier resolution. The classifiers are often used without an adaptation of the input resolution to the resolution of the specific database or camera which, unfortunately, is a common practice, especially in the solutions with a complicated structure of the classifier. An example of a quite complicated structure of the pedestrian detector is a deep convolutional neural network (CNN) [3,12,13,14,15]. In the case of the CNN, any change in the resolution of the CNN input layer causes necessity of adaptation in the other layers. It is quite complicated and therefore designers try to omit it. For example, in the proposed deep CNN by Kim, et al. [15], various grayscale pedestrian images were resized (mainly upscaled, as the smallest pedestrians had 50 pixels in height only) and colorized (!) to fit to the input size of the typical, pre-trained model of the CNN detector, which required 224 × 224 pixels and the color RGB input image format. Such solutions, although simple in implementation, are greatly ineffective.

In this context, we should be emphasize that high-resolution classifiers often, but not always, offer slightly higher detection performance, but always impose additional computational overhead. In fact, formally increasing the pedestrian candidates’ resolution does not increase the information content. Magnifying the subject also causes harmful blurring of the image and may have a negative effect during the classifier training. As a result, this can reduce efficiency, especially when there are largely disproportionate pedestrian samples in the training set. Finally, it is important to adjust the classifier resolution to that of the camera.

For the best results with a single classifier, we propose to search for a compromise image resolution. Therefore, we prepared and tested a number of classifiers with various resolutions. In the experiments, we decided to use three various baseline detectors, namely: histogram of oriented gradients (HOG) with the support vector machine (SVM) classifier, the aggregated channel feature (ACF) detector, and the deep convolutional neural network (CNN). The first two are commonly used in the standard real-time applications for the detection of pedestrians in IR images [9,10,16,17,18,19,20]. Recently, we have observed a rapid and promising development of various classifiers and detectors based on CNNs. They are very accurate but are quite computationally demanding and for real-time processing need specialized hardware, e.g., graphic processing units (GPUs) or tensor processing units (TPUs) [11,13,14,21,22].

For a fair comparison of results with various classifiers, we proposed a novel and universal performance index. We took the speed of detection and the classification accuracy into account. Using this index, we are able to select the best image resolution for a particular classifier.

The presented approach is quite general, i.e., it is applicable not only to the considered problem, but also to the detection of any type of object with any classifier.

To get a number of experiments, which is statistically sufficient, we selected a set of customized infrared night and day vision datasets, namely: CVC-09 (Computer Vision Center, FIR Sequence Pedestrian Dataset) [23], NTPD (Night-time Pedestrian Dataset) [24], LSI FIR (Laboratorio de Sistemas Inteligentes, Intelligent System Lab Far Infrared Pedestrian Dataset) [25], OSU (Ohio State University, Thermal Pedestrian Dataset) [26]. We selected these datasets for their versatility in IR technologies, image resolution, and environmental conditions. Moreover, the use of these publicly available datasets made it possible to compare our results fairly. We balanced statistically the datasets in terms of the numbers of positive and negative images (pedestrian wise).

The paper is organized as follows. After the introduction, we present an analysis of classification methods used for detection of pedestrians. Then, we introduce the proposed performance index. Next, we describe video datasets used in our experiments and methods for tuning of the considered classifiers. Consequently, we present the obtained results with their detailed evaluation. Finally, we show that, with the proposed performance index, it is possible to determine the optimal resolution for classification. The paper ends with some conclusions.

## 2. IR Systems for Detection of Pedestrians

Two infrared vision systems categories, i.e., passive and active can be distinguished. Passive systems detect electromagnetic radiation with wavelengths in the range of 3–30 μm. They are often called FIR (far infrared) systems [27]. Human bodies with temperatures of around 300 K have the highest energy emission in this band. As a result, the FIR images have a high contrast between living creatures and the environment [28]. Passive systems also provide a large detection range for high-quality cameras (up to 300 m). In addition, modern (cold) light sources do not dazzle passive IR cameras. However, on the other hand, these cameras are characterized by lower resolution and higher costs than cameras used in active IR systems and are more sensitive to changes in thermal contrast (according to the season, weather, humidity, etc.).

Active systems detect infrared light near the visible range (0.8–1.1 μm) and are therefore called NIR (near infrared) systems. They provide high-resolution images that are easy to interpret for humans due to the proximity of the NIR range to visible light. They are widely used due to the relatively low cost of cameras and their small size, especially in closed circuit television (CCTV). However, they have a limited detection range and require additional illuminators [4]. NIR detectors can also be dazzled by oncoming vehicle headlights (or illuminators) and perform much worse than the FIR ones in fog.

It should be stressed that images produced by NIR and FIR technologies differ a lot, thus the video processing stages, although in both cases process IR images, should be optimized separately.

In this paper, we present new ideas on selection of the optimal parameters for pedestrian classification regardless of the type of IR images.

### 2.1. General Pedestrian Detection Procedure

**Figure 1:**
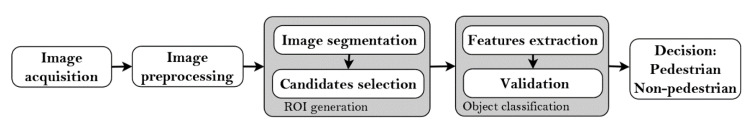
General pedestrian detection scheme.

Figure 1 presents a general scheme of the procedure for the pedestrian detection. Its first stage is the IR image acquisition and preprocessing. Then, in the second stage, the ROIs are generated, which should cover all areas with pedestrian candidates for further processing. The next stage is segmentation for the separation of pedestrians from the background (or more precisely the desired areas of the IR image that potentially contain pedestrians). Correctly segmented ROIs contain all objects to be detected (pedestrians), but together have as few other objects (none pedestrians) as possible. By such means, the amount of data that is transferred to the next stages is reduced [10,29]. There are plenty of solutions for proposing the pedestrian candidates, starting from the sliding window approach in a multi-scale manner [9,10] up to more faster and intelligent solutions [8,12,30], e.g., the specialized region proposal networks [31,32].

After the ROI generation, we realize the next, namely the pedestrian classification stage. This is crucial as it strongly affects the final quality of the pedestrian recognition [33]. In our opinion, the main role in the improvement of effectiveness of the overall system involves just optimization of this stage, regardless of which ROI generation technique is used.

In the simplest case, the classifier window exactly fits the original pedestrian area, but in practice, it is almost impossible.

### 2.2. Object Classification

The object classification stage consists of two steps: feature extraction and final validation with the selected classifier. The feature extraction step brings the most valuable features and reduces the amount of data that describes the object. In a validation step the classifier finally decides which objects are pedestrians and which are not. Finally, we calculate the performance index of the classifier.

To better present the problems regarding the classification stage, below, we present details about these two steps.

#### 2.2.1. Features Extraction

The feature extraction step is made in order to find the most valuable features and to reduce the amount of data that describes the object. There are many efficient feature extractors used for detection of pedestrians, starting with the basic handcrafted features like histograms of oriented gradients (HOG) [34], local binary patterns (LBP) [35], shape context [36], 1D/2D Haar descriptors [37], to plenty of their modifications [17,19,30,36,38]. Recently, several efficient variants of the HOG were proposed: integral channel features (ICF), for which the HOG descriptors are used together with luminance and UV chrominance components (LUV) [39], the ACF [40] combining HOG channel feature with the normalized gradient magnitude and LUV color channels, and the Checkerboards [41], which are modifications of the ICF. They perform filtering of the HOG+LUV feature channels. The listed feature extractors have become the state-of-the-art approaches for the night vision pedestrian detection [6].

Contrary to the mentioned handcrafted features, CNNs are now very strongly developed and widely used. The most important CNN models are: AlexNet/CaffeNet [42,43], VGG [44], ResNet [45]. They allow for self-learning of features and perform significantly better than other approaches. On the other hand, due to their complex structure, they need GPUs for real-time computations or operate much slower.

#### 2.2.2. Validation (Classifiers)

As mentioned in the introduction, a typical approach to the validation process is the use of a single classifier with a fixed window size. In result, all pedestrian candidates must be resized to the classifier resolution before the validation process can start [3,10,12,29].

The most popular classifiers are: SVM, AdaBoost (used in the ACF detector), neural networks (including: matrices of neurons, self-organizing maps [46,47], deep CNNs) and various combinations of them. Guo, et al. [48] used AdaBoost classifier for initial selection while the SVM classifier—for the final verification. Wang, et al. [49] proposed a combination of the AdaBoost with the random vector functional link neural network. Kong, et al. [50] proposed a parallel connection of various classifiers, trained in a complementary manner to each other. The result was high accuracy but low speed.

Liu, et al. [10] proposed a three-branch structured SVM classifier based on HIK (histogram intersection kernel). They achieved an increased performance of the detection for various heights of pedestrians. We have also deeply investigated this interesting technique [10] and expanded it to a form of multi-branch classifiers [51].

Jeon, et al. [52] presented and trained a combined classifier for various pedestrian poses, which was composed of four independent AdaBoost classifiers.

The classification part in the CNN models can be realized with fully-connected layers, however others solutions are also used [30]. Hou, et al. [13] utilized the deep neural network for classification purposes using multispectral information. Park, et al. [14] used CNN together with the AdaBoost classifier.

For the experiments, we decided to use three baseline detectors: HOG + SVM, ACF, CNN (our modification of the AlexNet/CaffeNet model similarly as in papers [3,12]).

## 3. Tuning Object Classification with Performance Index

As we stressed in previous sections, the classification stage is one of the crucial parts in the pedestrian detection procedure. Especially in the real-time applications with embedded systems (e.g., in cars) this stage must be fast and accurate. Therefore, in order to obtain optimal results, we propose to search for a compromise image resolution between the speed and accuracy.

Consequently, after a series of many experiments we propose in this paper a concept for comparing the results by introducing a novel and universal performance index

(1) $$\eta_{\mathrm{FPS}}=w\cdot a+\left(1-w\right)\cdot \mathrm{FPS},$$

where w∈〈0,1〉 weights the overall accuracy a and (1−w) weights the processing speed expressed in frames per second (FPS). By this means, we can control importance of accuracy versus FPS when designing the system. This is because, using this performance index, we are not only able to evaluate classifiers but also to select the best image resolution for a particular classifier taking the camera specificity (image resolution, camera type) into account.

Very often, during the design process, we assume that the processing speed is measured in FPS as it is a very important factor in real-time, especially in embedded systems. It characterizes the algorithms used, the computational platform, and finally the computation costs.

However, a direct use of the real FPS values makes the performance index related to the speed of the used computational platform (both hardware and software wise). That is why, in order to omit this drawback, instead of the real FPS parameter, we propose to use the relative value $t_{\mathrm{cal}}^{-1}/t_{\min}^{-1}$, where $t_{\mathrm{cal}}$ is the mean calculation time of one test sample with a given resolution (the time for extraction of the HOG features plus the classification time) and $t_{\min}$ is the minimum calculation time achieved over all possible resolutions. Therefore, the final, practical version of the proposed normalized performance index formula is as follows:

(2) $$\eta_{n}=w\cdot \frac{a}{100}+\left(1-w\right)\frac{t_{\min}}{t_{\mathrm{cal}}}.$$

Thus, both a/100 and $t_{\min}/t_{\mathrm{cal}}$ remain in the normalized 〈0,1〉 range.

**Figure 2:**
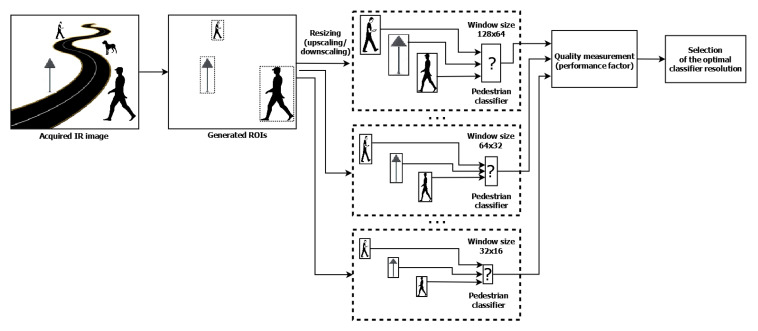
Processing scheme for tuning pedestrian classification with proposed performance index.

Figure 2 shows the resulting processing scheme with the proposed tuning procedure of the pedestrian classification process using the introduced performance index. There are the same processing stages in this scheme as those in Figure 1, i.e., acquisition of the IR image at the input, ROI generation, and pedestrian classification. To tune the classifier and perform tests with various image resolutions, after generating the ROI we resize all generated objects (by upscaling or downscaling them) to many various resolutions to match with the resolution of the classifier. We propose to adapt these resolutions starting with 64 × 128 to 16 × 32 in 13 steps. Then, we measure the classifications quality with the proposed performance index. Finally, by comparing the results, we can select the best resolution of the classifier for the given input data.

In the literature concerning the machine learning, we found many parameters describing the classifier effectiveness like: sensitivity, miss rate, precision, F1 score, etc. [53]. However, in our case, as we explain below and discuss in Section 5, only our suggestion, i.e., the weighted arithmetic mean, is the proper approach (Expressions (1) and (2)).

## 4. Experiments

**Figure 3:**
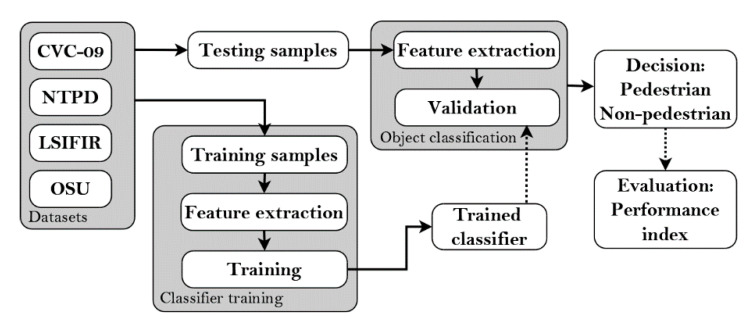
Test bed for comparison of tested classifiers.

In order to find the best resolution of the classifier applying the proposed performance index, we performed many experiments with various scenarios and using various night-vision video datasets containing pedestrians. For this purpose, we built a special test bed (Figure 3). In the experiments, we particularly checked an impact of the image resolution, classifier type, and the resulting number of features on the classification accuracy and the computation time, using three detectors, namely: HOG + SVM, ACF, and the deep CNN model.

### 4.1. Night Vision Pedestrian Datasets

The chosen night vision pedestrian datasets, differing in resolutions, qualities, and the acquisition techniques, are commonly used for benchmark tests in many papers [5,6]. They are: CVC-09 (Computer Vision Center, FIR Sequence Pedestrian Dataset), NTPD (Night-time Pedestrian Dataset), LSI FIR (Laboratorio de Sistemas Inteligentes, Intelligent System Lab Far Infrared Pedestrian Dataset), OSU (Ohio State University, Thermal Pedestrian Dataset) [9,23,25]. From all these datasets, we extracted ROI samples for both training and testing (Table 1).

#### 4.1.1. CVC-09 Thermal Pedestrian Dataset

The CVC-09 (Computer Vision Center, FIR Sequence Pedestrian Dataset) consists of two subsets of pedestrian thermal images: 5990 images recorded during days and 5081 images recorded at night. Their resolution is quite high as for the IR recordings and equals 640 × 480 pixels. The authors of this dataset inform that it was produced with the FIR thermal imaging technology, however, they do not specify the camera type and the temperature scale [23]. We concluded from our tests that the images have some unknown static temperature scale and that there is no contrast enhancement applied.

This dataset is very demanding as pedestrians occur with various sizes. Images recorded on days have low contrast between pedestrians and the background. This differs from other typical FIR recordings.

**Figure 4:**
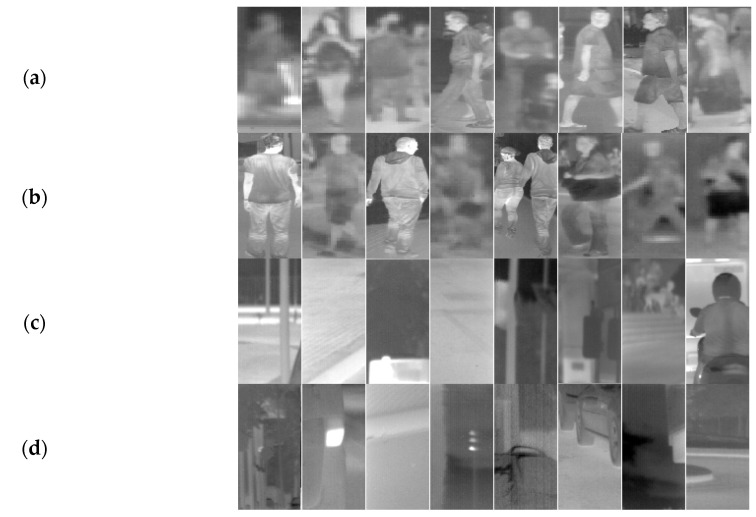
CVC-09 dataset of pedestrians: (**a**) day-time positive samples, (**b**) night-time positive samples, (**c**) day-time negative samples, (**d**) night-time negative samples.

We prepared the dataset with positive samples by clipping pedestrians out of the original images (Figure 4). The resulting dataset was annotated automatically. Therefore, there are some inaccuracies, e.g., not all pedestrians were correctly marked (cf., Figure 4b—in fact, a figure in the third column contains parts of two pedestrians).

**Figure 5:**
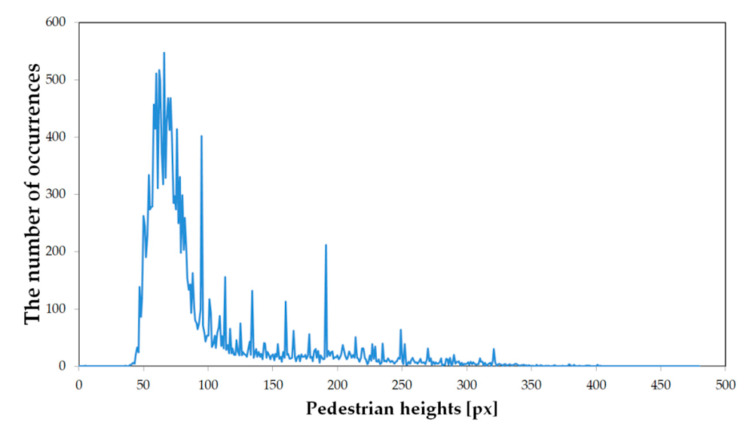
Distribution of pedestrian heights (in pixels) in CVC-09 dataset.

Due to the variety of distances between the camera and pedestrians, the obtained positive samples have different resolutions (from 3 × 6 up to 190 × 458 pixels). The height distribution of these samples is shown in Figure 5. Because all samples have to be scaled to a given classifier resolution, they sometimes must be significantly enlarged (up-scaled) and then they can be quite strongly blurred (cf., Figure 4).

The dataset with negative samples was prepared by cutting out chosen areas with no pedestrians. They were extracted with the window equaled to the size of the largest used classifier resolution (i.e., to 64 × 128 pixels). During the classifier training, the negative samples were then scaled down again to the required resolution. The prepared dataset is large enough for the statistical analysis. We offer it as an extension of the original dataset in our website [54].

#### 4.1.2. NTPD

**Figure 6:**
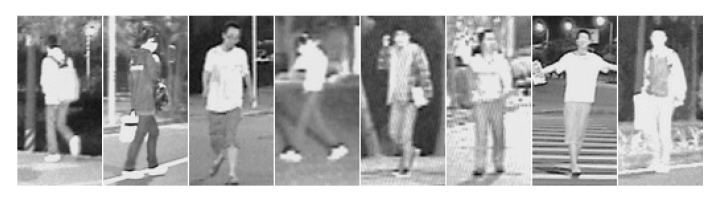
NTPD dataset pedestrian (positive) samples.

The NTPD (Night-time Pedestrian Dataset) [24] is divided into two sub-sets: training and testing. It consists of images of pedestrians stored with the NIR active system of resolution 64 × 128 pixels (cf., Figure 6). In this dataset, to make the classification process realistic, we extended the number of the negative samples similarly to those occurring in real situations of the automotive applications as an asymmetric distribution (much more negative samples than the positive ones) is quite typical. These negative samples were extracted from images, which contain no pedestrians. We also offer them in our website [54].

#### 4.1.3. LSI FIR Pedestrian Dataset

In the LSI FIR (Laboratorio de Sistemas Inteligentes/Intelligent System Lab Far Infrared Pedestrian Dataset) [25] the FIR images were acquired in outdoor urban scenarios. The images are divided into two subsets: the classification dataset and the detection dataset. In the first one the images are scaled to 32 × 64 pixels and include positive and randomly sampled negative images. The second one includes annotated original positive and negative images of 164 × 129 pixels resolution. In the experiments, we only used the first subset.

#### 4.1.4. OSU Thermal Pedestrian Dataset

**Figure 7:**
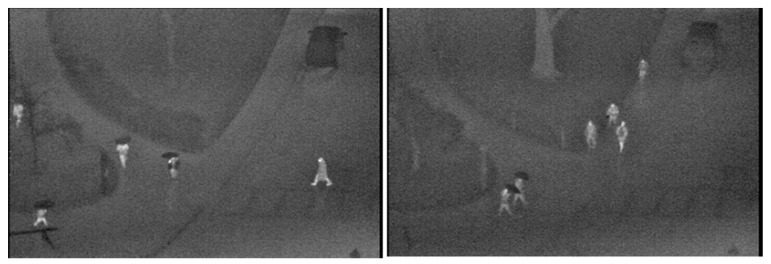
Two illustrative images from OSU dataset.

The OSU (Ohio State University) Thermal Pedestrian Dataset consists of 10 daytime video sequences captured on a university campus under various weather conditions (cf., Figure 7). These sequences were recorded using a passive thermal sensor Raytheon 300D [26]. Thus, the images have a resolution of 320 × 240 pixels.

On the basis of this dataset, several authors created their own, not standardized training and test subsets [6], but with a small number of samples. We also decided to prepare our own dataset from the OSU dataset [26]. Since pedestrians in the original dataset have low resolution, we decided to extract samples with a resolution of only 32 × 64 pixels. From a half of the images, we selected pedestrians who, together with their mirror images (used to increase the number of samples), formed positive training samples. From the second half of the images in the same way we created the training samples. To obtain negative samples (those without pedestrians), frames with the background only were cut with a window of size 32 × 64 pixels with spacing of 8 pixels. Additionally, their number was increased by rotation and mirroring vertically and horizontally. Finally, we obtained 3864 negative samples. A half of them was used for training and the other half for testing. The extended version of this database is also available in our website [26].

### 4.2. Classifier Training

The numbers of training and test samples in the prepared night vision datasets are quite varying, but statistically sufficient to conduct relevant experiments. All the prepared datasets are intentionally unbalanced as they have much but realistically more negative samples than the positive ones. This is because such relation is typical in reality for the target application (i.e., detection of pedestrians from a car at night, where images with no pedestrians occur much more often than those containing pedestrians). This however can lead to problems with the proper training of the classifier. If the classifier is trained to achieve the lowest possible learning error, this can lead to some reduction in the false positive rate. This is related to the greater number of negative slack variables that affect the objective function. To properly train the classifier with unbalanced data, in both data classes we should weigh samples as follows

(3) $$C_{1}=w_{1}C,\;\;C_{2}=w_{2}C\;\;\mathrm{with}\;w_{1}+w_{2}=1,$$

where C determines importance of the misclassification and is the Lagrange multiplier upper bound, used as the penalty parameter [47].

### 4.3. Resolution of the Classifier

**Figure 8:**
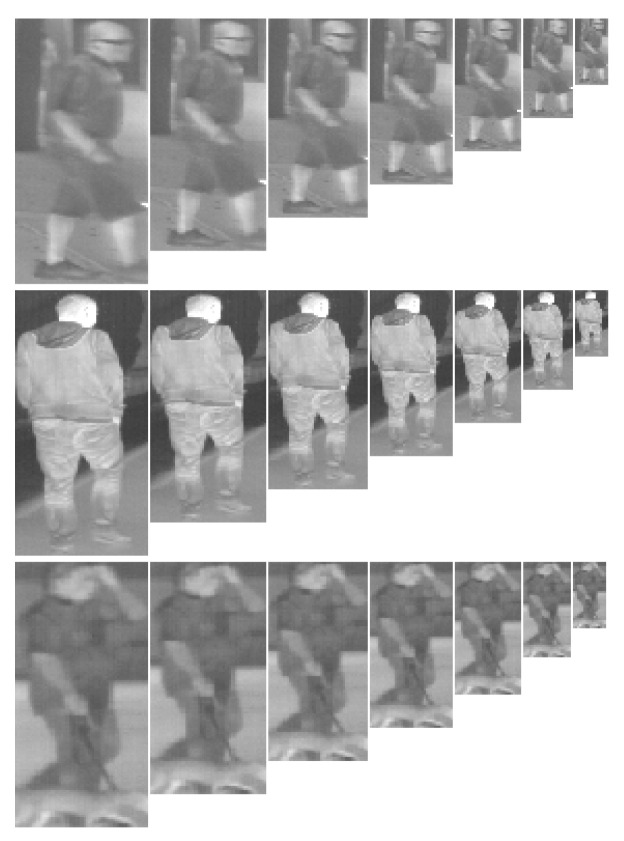
Three positive samples in various resolutions: 64 × 128, 56 × 112, 48 × 96, 40 × 80, 32 × 64, 24 × 48, 16 × 32; original images are in the CVC-09 dataset.

To perform experiments with different resolutions of the classifier, the initial images were scaled into several sizes: 64 × 128, 56 × 120, 56 × 112, 56 × 104, 48 × 96, 40 × 88, 40 × 80, 40 × 72, 32 × 64, 24 × 56, 24 × 48, 24 × 40, 16 × 32 (cf., Figure 8). From all of them, we formed 13 sets of testing images. These sets were prepared separately for individual datasets.

As we mentioned in Section 4.1.1, the CVC-09 dataset, has the pedestrians captured with many different sizes. In consequence, the initial resolutions varied a lot: from 3 × 6 pixels up to 190 × 458 pixels. To match these resolutions to the resolution of the classifiers, we scaled each image into the closest resolution of someone from the 13 listed above resolutions. Due to a relatively large span of the assumed classification resolutions, most of the images required slight scaling only.

On the other hand, in the rest of the analyzed datasets the initial resolutions were fixed. In the NTPD dataset it is 64 × 128 pixels, whereas in the LSI FIR and OSU datasets it is 32 × 64 pixels (after extraction, cf., Section 4.1.1). We assumed that the images were scaled down only (scaling up brings no additional information, but complicates the calculation, thus it is irrational). Finally, from the NTPD dataset we got 13 test sets, while from each of the LSI FIR and OSU datasets we prepared 5 test sets (numbered from 9 to 13).

### 4.4. HOG+SVM and ACF Detectors Configuration

While the resolution of images in different sets was varying, the rest of parameters for the HOG feature extractor was kept constant. For all test sets the number of bins was set to 9, block size to 16 × 16 pixels and the cell size to 8 × 8 pixels. For the SVM classifier, the linear kernel was used.

The ACF detector was implemented similarly as presented in [6]. In the case of night vision and gray-scale images (both passive and active ones) we adopted the ACF to have 8 feature channels: 6 HOG orientation bins, one normalized gradient magnitude and one luminance channel (instead of three LUV color channels). The AdaBoost was used as a classifier in the ACF detector to train and combine 2048 depth-two trees.

For both feature extractors, various resolutions strongly affect the number of features, which have to be analyzed by the classifier (cf., Table 2).

### 4.5. AlexNet/CaffeNet CNN Configuration

The original AlexNet/CaffeNet CNN architecture [42,43] was prepared for images of 224 × 224 resolution. This architecture is often used for classification purposes [3,12]. We adapted it to the lowest tested resolution, i.e., to 16 × 32 by reducing the size of the convolutional filters and the size of the maximum pooling. Our structure was identical for all tested image resolutions (to ensure fair comparison between them). Table 3 presents the details.

According to the image resolution the number of CNN parameters is very high and varies from ca. 7 million to more than 38 million (cf., Table 2).

### 4.6. Classification Accuracy and Calculation Time

At the beginning of the tests, we used the CVC-09 dataset, because, in our opinion, it presents a very similar material to that occurring in real situations. The images were taken during the day and in the night. The pedestrian regions have various sizes and therefore the analyzed ROIs have various resolutions. Next, we performed tests with the NTPD, LSI FIR, and the OSU datasets (the OSU results are placed in the bottom part of Table 4).

**Figure 9:**
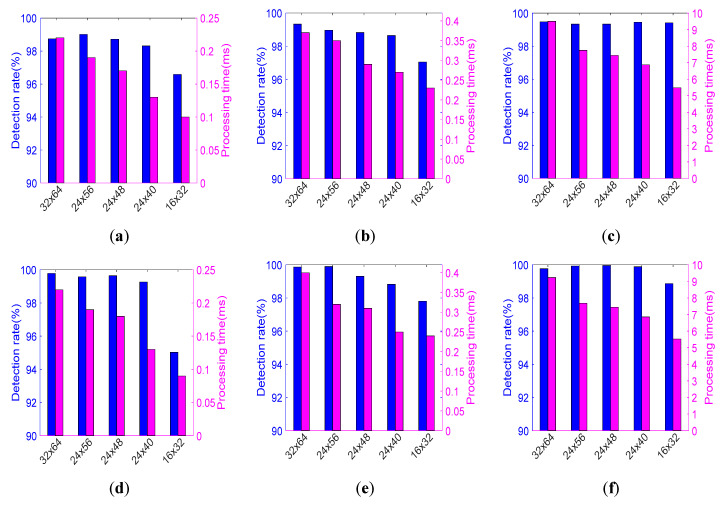
Detection rate and processing time as functions of image resolutions: HOG + SVM classifier (left column), ACF detector (middle column), CNN (right column) for the following datasets: LSIFIR (first row: **a**–**c**), OSU (second row: **d**–**f**).

**Figure 10:**
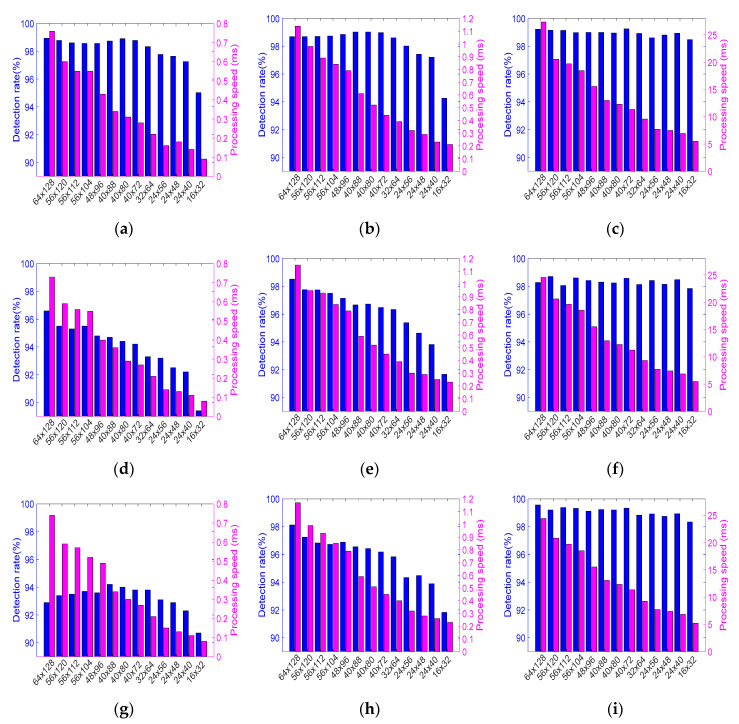
Detection rate and processing time as functions of image resolutions: HOG+SVM classifier (left column), ACF detector (middle column), CNN (right column) for the following datasets: NTPD (first row: **a**–**c**), CVC-09 night-time (second row: **d**–**f**), CVC-09 day-time (third row: **g**–**i**).

The obtained results are described in detail below, listed in Table 4 (which has the following columns: dataset, set name, frame size, classification accuracy, and calculation time), and are presented in Figure 9 and Figure 10. For each test set, we calculated the classification accuracy (referred also to as the pedestrian detection rate) and the mean calculation time.

The calculated detection accuracy values constitute points with equal false and miss detection probabilities. These points were computed with 180 test samples (90 positive and 90 negative).

The determined mean calculation times are composed of two phases: durations of the feature extraction process and times needed for classification of a single test sample. The processing was implemented in the C# programming language with EmguCV v. 2.4.10 environment [55] and LIBSVM [56] as the SVM library. The CNN was implemented with TensorFlow using Python language. The training process was performed with the GPU support in the Google Colab cloud environment. The usage of GPU allows parallelization of processing and therefore substantial speed-up of processing, but it strongly depends on many factors like the algorithm and data structure, or an architecture of the GPU. Therefore, in this paper the computations during the classification stage were made with a single CPU core to make fair, hardware independent comparisons between various methods and image resolutions. The following hardware was used: CPU Intel Core i7-6950X, GPU NVidia Quadro 2000 1GB, 8 GB of RAM.

### 4.7. Discussion on Results

The best detection accuracy was achieved with the CNN approach, but other results are also fully acceptable (cf., Table 4 and Figure 9 and Figure 10). In Table 4 we highlighted the results of classification accuracy, which are the best in the set of various resolutions of a given dataset and those which are close to the maximum values, but obtained with lower resolutions. It should be noticed, that in almost all cases (especially for the CNN) the results are good even for a quite low-quality input data.

For example, for the resolution of 24 × 40 the accuracy is almost as high (99.89%) as for the highest resolution among all datasets. Furthermore, for the CVC-09 daytime and NTPD datasets, the best accuracy is obtained for a lower resolution (40 × 72) than the maximum 64 × 128. The right columns of Figure 9 and Figure 10 show that for the CNN detector the graphs of the detection accuracy are almost flat.

The resolution of a sample strongly affects the processing time. It is true for all the classifiers. The CNN is the slowest solution (more than 20 times slower than the HOG+SVM or the ACF detector). For low resolution samples (e.g., 16 × 32) it needs ca. 5.5 ms and for high resolution ones (e.g., 64 × 128) it needs ca. 25 ms to calculate the result. The ACF detector is slightly slower than HOG+SVM, but achieves higher accuracy, especially for the CVC-09 and NTPD datasets. For processing low resolution samples (e.g., 16 × 32) the HOG+SVM detector needs 0.08 ms only, while ACF needs 0.21 ms. For high resolution samples (e.g., 64 × 128), the HOG+SVM needs about 0.75 ms to calculate the result while the ACF needs 1.15 ms (cf., Table 4).

Taking into account differences between the datasets, we see that in case of the CVC-09 datasets, the obtained detection accuracy values are very good (above 90%) for all tested detectors (cf., Table 4, Figure 9 and Figure 10). It can also be seen that in the day-time subset of the CVC-09, high effectiveness can be achieved with the HOG+SVM detector with a relatively low resolution of samples, i.e., just 40 × 88. The ACF detector achieves local optima with the resolution of 48 × 96. For the night-time subset of the CVC-09 dataset, both detectors (i.e., SVM and ACF) achieve mild local maxima of effectiveness with the resolution of samples equal to 56 × 104 (cf., Set 4 in Table 4 and Figure 10). For the night-time sets the detectors achieve better results than those for the day-time sets. It is due to the fact, that the thermal contrast at night is higher (cf., Figure 4a,c). During the analysis of other datasets (i.e., NTPD, LS IFIR, and OSU) the values of the obtained detection effectiveness are better than those for the CVC-09 dataset (all of them are above 95%, in many cases larger than 98%). It is valid for all resolutions (even very low) and for all classifiers. For the LSI FIR and OSU datasets the classification with the resolution equal to 24 × 48 achieves similar accuracies to the best ones, but with approximately 20% shorter time than this for the initial resolution (cf., Figure 9). For the NTPD dataset, the classifier resolution can be reduced to 40 × 80 while the effectiveness remains almost unchanged. By this reduction, the classification time is shorter by approximately 60% (cf., Figure 10).

Concluding, we see that the classification effectiveness does not diminish significantly, even if the image resolution substantially decreases. The upper limit of the classifier error is related to the dimension of the features vector and to the number of the training samples (therefore, we tried to maximize the training set) [47]. This relation is visible in our experiments (cf., Table 4, Figure 9 and Figure 10). Thus, we can state that, in general, the resolution of the classifier can be lower than the original resolution of the analyzed images. However, the best resolution should be obtained with the use of the proposed performance index.

## 5. Performance Index Results

**Figure 11:**
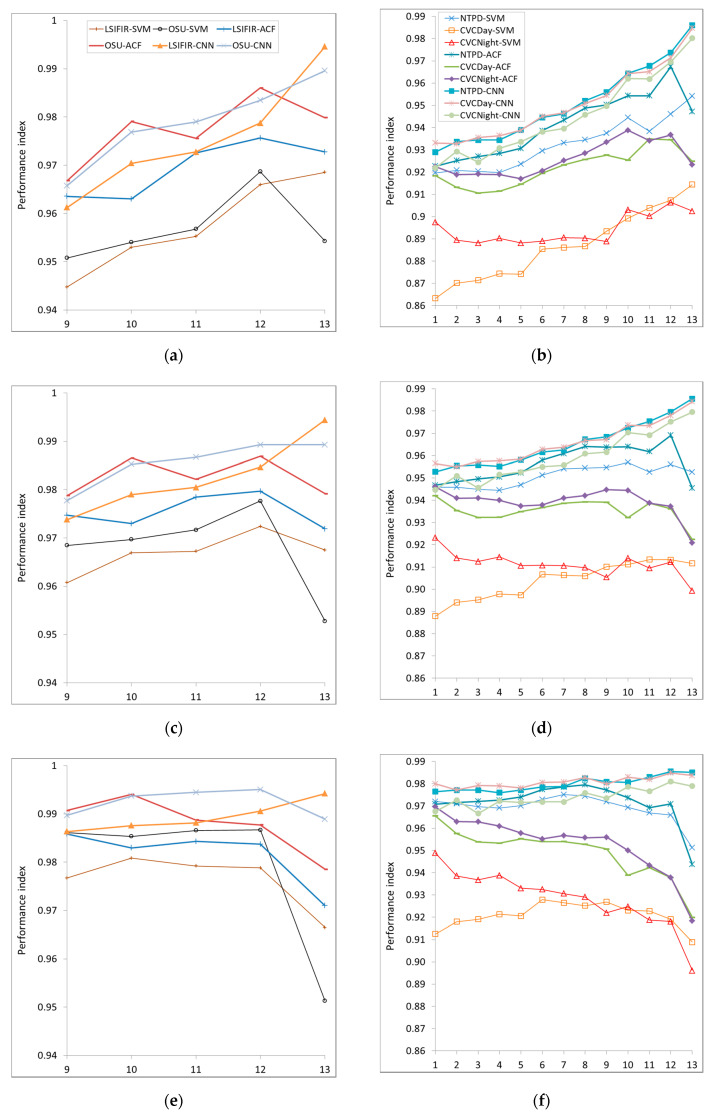
Performance indices as functions of image resolutions (values on *x*-axis refer to particular test sets in Table 4): for (**a**,**b**) w = 0.92, for (**c**,**d**) w = 0.95, for (**e**,**f**) w = 0.98, with w being the weight of accuracy for various datasets and classifiers indicated with different colors as explained in the legend.

Using the results of our experiments (Table 4) and Equation (2), we calculated the performance indices η for all datasets, resolutions, and tested classifiers. The results are presented in Figure 11, where values in the *x*-axis refer to the testing sets presented in Table 4.

As already mentioned in Section 3, the weighted sum of the relative accuracy a/100 and the inverse of the relative time $t_{\min}/t_{c}$ is the only proper approach to define the appropriate performance index η.

We noticed from experiments presented in Section 4.6 that accuracy a in formula (2) is greater than 90% for almost all configurations (cf., Table 4), whereas $t_{c}^{-1}/t_{\min}^{-1}$ varies in a large extent. In addition, we took into account the type of detection system. In our case, i.e., the pedestrian detection, it should be very accurate. Therefore, the variation of accuracy should have significantly higher influence on the impact factor than variation of the mean calculation time.

We proposed three values of the weight w for the performance index, depending on the application. We selected these values experimentally and adjusted as closely as possible to the three proposed application scenarios.

First, if the processing time is assumed to be very important, e.g., in applications with low power processing units like mobile platforms, the weight should be set to ca. w=0.92 (Figure 11a,b). In result, the performance index is higher for low object resolutions.

Second, if the accuracy is assumed to be much more important, e.g., for offline processing of CCTV recordings or safety and security systems, the weight w should be set ca. to w=0.98 (Figure 11e,f). In result, the performance index achieves the highest values for medium and high resolutions of the classifiers.

Third, for w=0.95 (Figure 11c,d), we get in some sense the balanced configuration, still with high accuracy importance, and taking changes in the processing time into account, e.g., in automotive and real-time security systems.

Most curves in Figure 11 have global and local maxima. We chose them to state the best performance resolutions for the tested classifiers. The results are collected in Table 5. Beside the best resolution, we present differences in processing accuracies and processing times (in percent), in relation to the classifier with the highest resolution. However, the decrease of accuracy varies from 0.14% to 2.55%, as the reduction of the processing time reaches more than 73% (cf., Table 5).

For some cases (as presented in Table 5), we achieved, by means of the resolution reduction, both the time reduction and the increase of the classification accuracy. Classifiers, which are tuned for the best performance index can process data up to four times faster with a slight decrease of the accuracy (merely by about 1−2%).

There is no universal best resolution for all cases, but the best performances are achieved for resolutions between 24 × 40 and 48 × 96 pixels (cf., Table 4, testing sets from No. 7 to 12).

## 6. Conclusions

In this paper, we tested and optimized the real-time object classification procedure to increase the pedestrian detection performance. Tests made using four different night and day IR vision datasets showed that the enlargement of the window size used for feature selection and classification does not always improve the classification accuracy, but it always requires more time for processing.

In general, the pedestrian detection is similar during the day and night. The main difference is the image segmentation process (executed before the classification stage). Therefore, the presented considerations are also applicable for the day-time recordings.

Our important achievement is a suggestion of the novel performance index for the evaluation of classifiers, which takes the speed of detection and the classification accuracy into account. With the proposed performance index, we selected the best resolution of the analysis window in a given dataset and classifier. With such newly reduced resolution, which is typically much lower than the initial resolution (that of the input images), we can decrease the processing time needed for the classification by up to 74% with insignificant influence on the accuracy.

For all tested detectors, i.e., HOG + SVM, ACF, and the adapted CNN, we were able to decrease the processing time without decreasing the classification accuracy. This confirms the versatility of the proposed method. It should be noticed that the CNN obtains the best accuracies for various databases, and the gains achieved with the input resolution tuning are the highest.

Last but not least, it should be stressed that the presented approach is quite general and can also be used to select parameters for many different classes of specified tasks of real-time classification of various objects (i.e., not only for pedestrians).

In future, we plan to prepare our own database, offer it to the interested community, prepare further evaluation experiments, and compare their results with those just presented.

## Figures and Tables

**Table 1:** Number of training and test samples in night vision datasets.

	No. of Training Samples		No. of Test Samples	
Dataset	Positive Samples	Negative Samples	Positive Samples	Negative Samples
CVC-09 FIR Day-time	11,839	25,410	6711	75,398
CVC-09 FIR Night-time	6998	30,030	7862	72,985
Extended NTPD	1998	8730	2370	12,600 (*)
LSI FIR	10,208	43,390	5944	22,050
OSU	1004	1932	964	1932

(*) Extended number of test negative samples in compare to [19].

**Table 2:** Number of features of HOG and ACF feature extractors, and number of parameters in the adapted CNN for various resolutions.

	Number of Features		Number of Parameters
Frame Size [px]	HOG	ACF	CNN
64 × 128	3780	4096	38,686,369
56 × 120	3024	3360	32,657,057
56 × 112	2808	3136	30,822,049
56 × 104	2592	2912	28,987,041
48 × 96	1980	2304	24,006,305
40 × 88	1440	1760	19,549,857
40 × 80	1296	1600	18,239,137
40 × 72	1152	1440	16,928,417
32 × 64	756	1024	13,520,545
24 × 56	432	672	10,636961
24 × 48	360	576	9,850,529
24 × 40	288	480	9,064,097
16 × 32	108	256	7,229,089

**Table 3:** Proposed CNN structure.

No.	Layer Type	Elements	Activation Function	Remarks
1	convolutional	48, 7 × 7 filters	ReLU	maximum pooling, filter size 2 × 2, local response normalisation
2	convolutional	128, 5 × 5 filters	ReLU	maximum pooling, filter size 2 × 2, local response normalisation
3	convolutional	192, 3 × 3 filters	ReLU	-
4	convolutional	192, 3 × 3 filters	ReLU	-
5	convolutional	128, 3 × 3 filters	ReLU	maximum pooling, filter size 2 × 2
6	fully connected	2048 neurons	ReLU	dropout ratio of 0.5
7	fully connected	2048 neurons	ReLU	dropout ratio of 0.5
8	output	1 neuron	sigmoid	pedestrian detection score

**Table 4:** Number of configuration sets, classification effectiveness for experimental datasets.

Dataset	Set	Frame Size [px]	Classification Accuracy (*) [%]			Calculation Time (**) [ms]		
			HOG+SVM	ACF	CNN	HOG+SVM	ACF	CNN
CVC-09 day-time subset	1	64 × 128	92.9	**98.12**	**99.56**	0.74	1.17	24.41
	2	56 × 120	93.4	97.24	99.20	0.59	0.99	20.79
	3	56 × 112	93.5	96.83	99.38	0.57	0.93	19.70
	4	56 × 104	93.7	96.72	99.32	0.52	0.85	18.48
	5	48 × 96	93.6	96.88	99.12	0.49	0.79	15.53
	6	40 × 88	**94.2**	96.55	99.24	0.34	0.59	13.05
	7	40 × 80	**94.0**	96.43	99.21	0.30	0.51	12.32
	8	40 × 72	93.8	96.18	**99.34**	0.27	0.45	11.35
	9	32 × 64	93.8	95.83	98.83	0.21	0.40	9.25
	10	24 × 56	93.1	94.34	98.92	0.15	0.32	7.71
	11	24 × 48	92.9	94.48	98.75	0.13	0.28	7.39
	12	24 × 40	92.3	93.89	98.93	0.11	0.26	6.83
	13	16 × 32	90.7	91.83	98.34	0.08	0.23	5.23
CVC-09 night-time subset	1	64 × 128	**96.6**	**98.53**	98.28	0.73	1.15	24.60
	2	56 × 120	95.5	97.77	**98.71**	0.59	0.95	20.62
	3	56 × 112	95.3	97.75	98.07	0.56	0.93	19.63
	4	56 × 104	95.5	**97.50**	98.61	0.55	0.84	18.54
	5	48 × 96	94.8	97.14	98.43	0.40	0.79	15.54
	6	40 × 88	94.7	96.67	98.31	0.36	0.59	12.97
	7	40 × 80	94.4	96.72	98.26	0.29	0.52	12.26
	8	40 × 72	94.2	96.48	98.59	0.27	0.45	11.25
	9	32 × 64	93.3	96.34	98.14	0.21	0.39	9.34
	10	24 × 56	93.2	95.38	98.42	0.14	0.30	7.72
	11	24 × 48	92.5	94.64	98.15	0.13	0.29	7.42
	12	24 × 40	92.2	93.82	**98.48**	0.11	0.25	6.88
	13	16 × 32	89.4	91.67	97.85	0.08	0.23	5.46
NTPD	1	64 × 128	**98.94**	98.69	**99.23**	0.76	1.14	27.37
	2	56 × 120	98.78	98.70	99.16	0.60	0.98	20.53
	3	56 × 112	98.61	98.71	99.14	0.55	0.89	19.70
	4	56 × 104	98.56	98.74	98.98	0.55	0.84	18.46
	5	48 × 96	98.57	98.85	98.99	0.43	0.79	15.55
	6	40 × 88	98.74	99.03	98.99	0.34	0.61	12.99
	7	40 × 80	**98.91**	**99.03**	98.96	0.31	0.52	12.29
	8	40 × 72	98.78	98.98	99.26	0.28	0.44	11.32
	9	32 × 64	98.34	98.61	98.92	0.22	0.39	9.58
	10	24 × 56	97.77	98.02	98.61	0.16	0.32	7.70
	11	24 × 48	97.65	97.43	98.81	0.18	0.29	7.50
	12	24 × 40	97.25	97.21	**98.94**	0.14	0.23	6.93
	13	16 × 32	95.02	94.26	98.48	0.09	0.21	5.50
LSI FIR	9	32 × 64	98.74	**99.33**	**99.47**	0.22	0.37	9.50
	10	24 × 56	**99.01**	98.96	99.33	0.19	0.35	7.75
	11	24 × 48	98.72	98.82	99.33	0.17	0.29	7.44
	12	24 × 40	98.31	98.64	99.45	0.13	0.27	6.87
	13	16 × 32	96.58	97.04	**99.41**	0.10	0.23	5.48
OSU	9	32 × 64	**99.79**	99.87	99.77	0.22	0.40	9.24
	10	24 × 56	99.58	**99.90**	99.93	0.19	0.32	7.69
	11	24 × 48	99.65	99.31	**99.96**	0.18	0.31	7.45
	12	24 × 40	**99.27**	98.83	99.89	0.13	0.25	6.86
	13	16 × 32	95.03	97.81	98.87	0.09	0.24	5.53

(*) The classification accuracy is a point on the DET curve with equal false alarm miss probabilities. (**) The presented mean calculation time takes a sum of the process of features extraction and classification of one test sample mean times into account.

**Table 5:** Configuration sets, classification accuracy and processing time for testing subsets.

Dataset	Type of Classifier	Best Performance Resolution	Difference in Accuracy (*) [%]	Processing Time Reduction (*) [%]
LSIFIR	SVM	24 × 56	0.27	−13.64
	ACF	24 × 40	−0.69	−65.56
	CNN	16 × 32	−0.06	−42.31
OSU	SVM	24 × 48	−0.14	−18.18
	ACF	24 × 56	0.03	−13.64
	CNN	24 × 40	0.12	−25.76
NTPD	SVM	40 × 72	−0.16	−63.16
	ACF	40 × 72	0.29	−61.41
	CNN	40 × 72	0.03	−58.64
CVC-09 Day-time	SVM	32 × 64	0.97	−71.62
	ACF	48 × 96	−1.06	−33.33
	CNN	24 × 40	−0.63	−72.02
CVC-09 Night-time	SVM	40 × 80	−2.28	−60.27
	ACF	32 × 64	−2.22	−66.09
	CNN	24 × 40	0.21	−73.83

(*) Percentage difference referred to the classifier with the highest resolution.
